# AN ANCHOR FOR BETTER PHYSICAL HEALTH AMONG OLDER ADULTS: POSITIVE FAMILY WELL-BEING

**DOI:** 10.1093/geroni/igae098.2776

**Published:** 2024-12-31

**Authors:** Yuchen Liu, Dae Hyun Kim, Eve Wittenberg

**Affiliations:** Harvard T.H. Chan School of Public Health, Boston, Massachusetts, United States; Hebrew SeniorLife, Boston, Massachusetts, United States; Harvard T.H. Chan School of Public Health, Boston, Massachusetts, United States

## Abstract

The decline in physical health among older adults corresponds to an increased need for social support and caregiving. In the Chinese context, where formal long-term care options are limited, families predominantly shoulder caregiving responsibilities. We hypothesize that the quality of these familial interactions could significantly influence older adults’ objective health. We used data from the China Health and Retirement Longitudinal Study, assessing family well-being through satisfaction with spouse and children, and measuring objective health via frailty index based on the accumulation of deficits. Our findings indicate that satisfaction with children or spouses is negatively associated with the accumulation of additional deficits, demonstrating a mean ratio (MR) of 0.80 (95% CI: 0.76-0.83) for children and an MR of 0.77 (95% CI: 0.74-0.80) for spouses. After stratifying the analysis by marital status, the association between satisfaction with children and frailty status revealed a more substantial effect size in single or unpartnered individuals (relative risk (RR): 0.74 CI: 0.68-0.81) compared to those who were married or partnered (RR: 0.81, 95% CI: 0.77-0.86). We speculate that those without a spouse or partner may rely more heavily on their children for physical support, thus developing a stronger bond and accounting for the observed larger effect size. In conclusion, positive family well-being correlates with better physical health. Implementing policies that offer financial assistance or incentives for family involvement in elderly care could mitigate financial strains and enhance health outcomes for older adults.

